# Comment to: The Outcomes of Delayed Revascularization in Lower Extremity Vascular Injury: A Retrospective Cohort Study

**DOI:** 10.5704/MOJ.2507.020

**Published:** 2025-07

**Authors:** E Boga

**Affiliations:** 1 Emergency Department, Esenyurt Necmi Kadioglu State Hospital, Istanbul, Turkey

Dear editor,

The article by Chai *et al*^1^, which appeared in the March 2025 issue of the Malaysian Orthopaedic Journal under the title “The Outcomes of Delayed Revascularization in Lower Extremity Vascular Injury” is one that I have read with much interest. This is a retrospective cohort study that sheds light on the results of the attempts at limb salvage in cases of prolonged ischemia, which is a common occurrence in the developing healthcare systems where patients usually come in late.

The study results are quite interesting given that a limb salvage rate of 89.8% was achieved even though the mean ischemic time was 14.1 hours and only 6 patients (10.2%) needed a secondary amputation. The popliteal artery was the most frequently injured vessel, and the majority of revascularizations were performed using contralateral saphenous vein grafts. More importantly, 83.1% of the patients had a Mangled Extremity Severity Score (MESS) of ≥7 and yet limb salvage was possible in the majority of the cases. This goes against the conventional wisdom that any score of MESS ≥7 should lead to amputation.

This study has a major advantage of looking at the components of the MESS score in terms of the level of ischemia and shock only as opposed to the overall score. Indeed, patients who needed an amputation had higher scores for ischemia (6.00 vs 4.79) and shock (1.00 vs 0.26) than the rest of the cohort suggesting that these components of the MESS may be more sensitive to limb salvage than the MESS as a whole. This is a major shift from the previous approach where decisions were made based on the score and not the patient’s individual case.

Furthermore, the study provides information on the renal complications of delayed revascularization. In this study, six patients developed acute kidney injury (AKI) that needed renal replacement therapy (RRT) but only one patient had to be put on lifelong dialysis. The average peak creatine kinase (CK) levels in rhabdomyolysis cases were over 11,000 U/L, which supports the use of CK as a marker. However, the absence of a statistically significant correlation between CK levels and RRT requirements indicates that CK should not be used on its own in the prediction of renal problems.

As for the recovery of function, 67% of the patients could walk on their own by the end of the first year of the study. However, 11.9% of the patients still used wheelchairs, which means that anatomical success does not always translate to the recovery of function. These results emphasise the fact that limb viability is not the only factor to consider in the management of patients and that long term mobility and quality of life should also be taken into account.

There are however certain limitations of the study. The retrospective, single centre design of the study and the absence of objective neurological assessment may limit the generalisability of the results. Also, the selection criteria included only non-viable limbs, which may have resulted in biased results in favour of better outcomes. However, the study provides strong evidence that delayed revascularisation – if carefully selected and performed in consultation with other specialists – can be done with acceptable results.

In conclusion, this study thus challenges the use of strict time limits and MESS score as the only criteria in the management of lower extremity vascular trauma. It advocates for a more sensitive and individualised approach to patient care, especially in resource-limited situations. I would like to thank the authors for their excellent contribution to the topic.
