# Supplementary material for: Comment to: The Outcomes of Delayed Revascularization in Lower Extremity Vascular Injury: A Retrospective Cohort Study
Source: Malays Orthop J. 2025 Jul;19(2):140–1. doi: 10.5704/MOJ.2507.020 (PMC12368444; doi:10.5704/MOJ.2507.020)
Supplement: Related texts

# Authors’ Reply to the Letters to the Editor

**Published:** 2025-07

**Authors:** Faisham WI, Mat-Saad AZ, Rosli MA, Chai KS, Wan-Sulaiman WA, Jusoh MH, Paiman M, Halim AS

Thank you for your comment. This article was written to share our experience and insights in managing delayed revascularization for lower limb vascular injuries^1^. This remains a common challenge in the Asia-Pacific region, where access to tertiary centres capable of performing advanced vascular surgery is often limited and geographically distant. We are pleased that one of our key messages - regarding the limitations of the Mangled Extremity Severity Score (MESS) in predicting amputation outcomes in severely injured limbs - has been clearly understood. We emphasise that MESS should not be used as a rigid indicator for clinical decision-making without a thorough assessment. Although this is a retrospective study, it reflects real-life clinical scenarios frequently encountered in various parts of the world, where potentially functional limbs can still be salvaged despite delayed intervention.

Our focus on delayed revascularization builds upon our earlier publication in 2014, which studied a similar patient cohort. In that retrospective review of 45 consecutive vascular injuries over seven years, more than half of the patients experienced delayed treatment. Notably, 73.3%were treated more than six hours post-injury, and six patients underwent revascularization beyond 24 hours^2^. We concluded that while early detection and intervention remain crucial, delayed revascularization can still result in acceptable outcomes. particularly in patients with adequate collateral circulation and no signs of ischemic neurological deficit. A critical point is that patients undergoing delayed revascularization, even in the absence of distal pulses must show good clinical collateral flow, confirmed by CT angiography with visible distal run-off.

Additionally, we have expanded our salvage efforts by employing extracorporeal perfusion using a cardiopulmonary bypass machine (CPBM) to prolong limb viability or allow for replantation after extended ischemia. This external shunting technique with CPBM can be safely performed to enhance limb survival and mitigate reperfusion injury, especially in patients who present late with prolonged ischemic time^3^.

We hope the findings of this study will reach a wider audience and serve as a turning point in the management of vascular injuries, encouraging more clinicians to consider limb salvage options even beyond the so-called “golden hours.”

## References

[1] Chai KS, Faisham WI, Wan-Sulaiman WA, Rosli MA, Mat-Saad AZ, Jusoh MH (2025). The Outcomes of Delayed Revascularization in Lower Extremity Vascular Injury: A Retrospective Cohort Study.. Malays Orthop J..

[2] Jagdish K, Paiman M, Nawfar A, Yusof M, Zulmi W, Azman W (2014). The outcomes of salvage surgery for vascular injury in the extremities: a special consideration for delayed revascularization.. Malays Orthop J..

[3] Wan Ismail  WFNB, Bin Wan Sulaiman  WA, Saad  AZBM, Mokthar  AMB,  Paiman  MB, Jusoh  MHB (2023). The heart-lung machine in major limb replantation: Report of two cases.. Microsurgery..
